# Supplementary material for: Developing Automated Computer Algorithms to Track Periodontal Disease Change from Longitudinal Electronic Dental Records
Source: Diagnostics (Basel). 2023 Mar 8;13(6):1028. doi: 10.3390/diagnostics13061028 (PMC10047444; doi:10.3390/diagnostics13061028)
Supplement: Supplementary file 1 [file diagnostics-13-01028-s001.zip › diagnostics-2142540-supplementary.pdf]

Supplementary materials

Table S1: Number of patients whose disease status did not change from their first visit to their last visit between June 1, 2005 and August 1, 2019.

From “disease stage” TO “disease stage”	Number of patients
generalized mild periodontitis to generalized mild periodontitis	38
generalized mild gingivitis to generalized mild gingivitis	729
generalized moderate periodontitis to generalized moderate periodontitis	725
generalized moderate gingivitis to generalized moderate gingivitis	439
generalized gingivitis to generalized gingivitis	274
localized mild periodontitis to localized mild periodontitis	215
generalized mild to moderate periodontitis to generalized mild to moderate periodontitis	140
generalized severe periodontitis to generalized severe periodontitis	90
localized moderate periodontitis to localized moderate periodontitis	84
localized mild gingivitis to localized mild gingivitis	80
generalized moderate to severe periodontitis to generalized moderate to severe periodontitis	73
generalized mild to moderate gingivitis to generalized mild to moderate gingivitis	48
generalized periodontitis to generalized periodontitis	42
localized gingivitis to localized gingivitis	39
localized severe periodontitis to localized severe periodontitis	34
localized periodontitis to localized periodontitis	30
generalized severe gingivitis to generalized severe gingivitis	29
generalized mild gingivitis to generalized mild periodontitis	27
localized mild periodontitis to generalized mild periodontitis	21
localized moderate gingivitis to localized moderate gingivitis	11
localized mild to moderate periodontitis to localized mild to moderate periodontitis	11
generalized moderate to severe gingivitis to generalized moderate to severe gingivitis	9
localized severe gingivitis to localized severe gingivitis	4
localized moderate to severe periodontitis to localized moderate to severe periodontitis	4
localized mild to moderate gingivitis to localized mild to moderate gingivitis	3

localized moderate to severe gingivitis to localized moderate to severe gingivitis	1
Total	3,919

Table S2: Number of patients whose disease status progressed from their first visit to the last visit between June 1, 2005 and August 1, 2019.

From "disease stage" TO "disease stage"	Number of patients
generalized mild periodontitis to localized moderate periodontitis	77
generalized moderate periodontitis to localized severe periodontitis	66
generalized mild periodontitis to generalized moderate periodontitis	56
generalized gingivitis to localized mild periodontitis	35
generalized mild to moderate periodontitis to generalized moderate periodontitis	26
generalized mild periodontitis to localized severe periodontitis	26
generalized moderate gingivitis to localized mild periodontitis	25
localized mild periodontitis to generalized mild gingivitis	24
generalized moderate gingivitis to generalized mild periodontitis	23
generalized mild gingivitis to localized moderate periodontitis	18
generalized mild periodontitis to generalized mild to moderate periodontitis	17
generalized mild gingivitis to generalized moderate gingivitis	15
generalized moderate gingivitis to generalized moderate periodontitis	15
localized mild gingivitis to generalized mild gingivitis	14
generalized mild to moderate periodontitis to localized severe periodontitis	14
generalized gingivitis to localized moderate periodontitis	13
generalized mild to moderate periodontitis to localized moderate periodontitis	11
localized mild periodontitis to localized moderate periodontitis	11
generalized mild gingivitis to localized severe periodontitis	10
generalized moderate periodontitis to generalized severe periodontitis	10
generalized gingivitis to generalized mild periodontitis	9
generalized moderate gingivitis to localized severe periodontitis	7
generalized gingivitis to localized periodontitis	7
localized mild periodontitis to localized severe periodontitis	6
localized mild periodontitis to generalized moderate periodontitis	6
generalized mild gingivitis to generalized moderate periodontitis	6
generalized gingivitis to localized severe periodontitis	6
generalized mild gingivitis to localized moderate gingivitis	6
generalized severe gingivitis to localized mild periodontitis	5
generalized mild gingivitis to generalized mild to moderate gingivitis	5

generalized moderate to severe periodontitis to localized severe periodontitis	5
generalized mild periodontitis to localized moderate to severe periodontitis	4
localized mild gingivitis to localized mild periodontitis	4
generalized moderate gingivitis to generalized mild to moderate periodontitis	3
localized mild to moderate periodontitis to generalized mild periodontitis	3
generalized mild to moderate gingivitis to localized mild periodontitis	3
generalized moderate gingivitis to generalized mild to moderate gingivitis	3
generalized mild periodontitis to localized mild to moderate periodontitis	3
localized moderate periodontitis to generalized moderate periodontitis	3
localized mild gingivitis to generalized moderate gingivitis	3
localized mild to moderate periodontitis to generalized moderate periodontitis	2
generalized mild periodontitis to generalized severe periodontitis	2
localized moderate periodontitis to localized severe periodontitis	2
generalized moderate to severe gingivitis to generalized moderate periodontitis	2
localized mild periodontitis to localized moderate to severe periodontitis	2
localized mild to moderate gingivitis to localized mild periodontitis	2
generalized moderate gingivitis to localized severe gingivitis	2
generalized mild to moderate gingivitis to generalized mild periodontitis	2
generalized mild to moderate periodontitis to generalized severe periodontitis	2
generalized moderate periodontitis to generalized moderate to severe periodontitis	2
generalized moderate periodontitis to localized moderate to severe periodontitis	2
generalized mild to moderate gingivitis to localized moderate periodontitis	2
generalized moderate to severe periodontitis to generalized severe periodontitis	2
localized mild to moderate gingivitis to generalized mild to moderate gingivitis	2
generalized severe gingivitis to generalized mild periodontitis	2
generalized mild to moderate gingivitis to generalized moderate gingivitis	2
localized mild periodontitis to localized mild to moderate periodontitis	2
generalized mild gingivitis to generalized mild to moderate periodontitis	2
localized gingivitis to generalized gingivitis	2
generalized mild to moderate gingivitis to localized mild to moderate periodontitis	2
localized gingivitis to localized periodontitis	1
localized moderate gingivitis to generalized moderate gingivitis	1
localized mild gingivitis to localized moderate gingivitis	1
generalized severe gingivitis to localized severe periodontitis	1
generalized moderate to severe gingivitis to localized mild to moderate periodontitis	1
localized moderate gingivitis to generalized moderate periodontitis	1
localized moderate gingivitis to localized moderate periodontitis	1

generalized mild periodontitis to generalized moderate to severe periodontitis	1
generalized mild gingivitis to generalized severe gingivitis	1
localized mild gingivitis to generalized moderate to severe gingivitis	1
localized mild periodontitis to generalized severe periodontitis	1
generalized moderate gingivitis to generalized severe gingivitis	1
generalized mild gingivitis to localized mild to moderate gingivitis	1
localized severe periodontitis to generalized severe periodontitis	1
generalized mild to moderate gingivitis to generalized moderate periodontitis	1
generalized severe gingivitis to generalized mild to moderate periodontitis	1
generalized moderate to severe gingivitis to localized mild periodontitis	1
localized mild gingivitis to generalized moderate periodontitis	1
generalized mild to moderate periodontitis to generalized moderate to severe periodontitis	1
localized severe gingivitis to localized mild periodontitis	1
localized mild periodontitis to generalized mild to moderate periodontitis	1
localized moderate to severe gingivitis to generalized moderate periodontitis	1
localized moderate to severe gingivitis to generalized moderate to severe gingivitis	1
generalized severe gingivitis to localized moderate periodontitis	1
generalized moderate gingivitis to generalized moderate to severe periodontitis	1
generalized mild to moderate gingivitis to generalized moderate to severe gingivitis	1
Total	669

Table S3: Number of patients whose disease status improved from their first visit to the last visit between June 1, 2005 and August 1, 2019.

From “disease stage” TO “disease stage”	Number of patients
generalized moderate periodontitis to generalized mild periodontitis	76
generalized mild gingivitis to localized mild periodontitis	50
generalized mild periodontitis to generalized mild gingivitis	32
generalized mild periodontitis to localized mild periodontitis	30
generalized mild gingivitis to localized mild gingivitis	20
generalized severe periodontitis to generalized moderate periodontitis	19
generalized moderate periodontitis to localized moderate periodontitis	19
generalized moderate gingivitis to localized moderate periodontitis	18
generalized moderate to severe periodontitis to generalized moderate periodontitis	18
generalized moderate gingivitis to generalized mild gingivitis	17

generalized mild periodontitis to generalized moderate gingivitis	15
generalized moderate periodontitis to generalized mild to moderate periodontitis	12
generalized moderate periodontitis to localized mild periodontitis	12
localized moderate periodontitis to generalized moderate gingivitis	11
generalized moderate periodontitis to generalized moderate gingivitis	10
generalized moderate gingivitis to localized mild gingivitis	10
generalized moderate periodontitis to generalized mild gingivitis	10
generalized severe periodontitis to localized severe periodontitis	9
localized moderate periodontitis to generalized mild periodontitis	8
localized No disease to localized No disease	7
localized mild periodontitis to localized mild gingivitis	7
generalized mild periodontitis to localized mild gingivitis	7
generalized mild periodontitis to localized moderate gingivitis	6
generalized moderate to severe periodontitis to generalized mild periodontitis	6
generalized gingivitis to localized mild gingivitis	5
localized moderate periodontitis to localized mild periodontitis	5
generalized moderate gingivitis to localized moderate gingivitis	4
generalized severe gingivitis to generalized mild gingivitis	4
generalized moderate to severe periodontitis to localized moderate to severe periodontitis	3
generalized severe periodontitis to generalized mild periodontitis	3
localized mild to moderate periodontitis to generalized mild to moderate gingivitis	3
generalized mild to moderate periodontitis to generalized mild to moderate gingivitis	3
generalized moderate periodontitis to generalized severe gingivitis	3
localized mild to moderate periodontitis to localized mild periodontitis	3
localized severe periodontitis to generalized moderate periodontitis	3
localized severe periodontitis to localized mild periodontitis	3
generalized mild to moderate gingivitis to generalized mild gingivitis	3
generalized severe periodontitis to generalized mild to moderate periodontitis	2
generalized mild periodontitis to generalized severe gingivitis	2
generalized mild to moderate periodontitis to localized mild periodontitis	2
generalized mild to moderate periodontitis to generalized moderate gingivitis	2
generalized moderate to severe periodontitis to generalized mild to moderate periodontitis	2
generalized mild to moderate periodontitis to generalized mild gingivitis	2
localized moderate to severe periodontitis to generalized moderate periodontitis	2

localized moderate to severe periodontitis to generalized mild periodontitis	2
generalized gingivitis to localized gingivitis	2
generalized mild to moderate periodontitis to generalized severe gingivitis	2
localized severe periodontitis to generalized mild periodontitis	2
localized moderate periodontitis to generalized mild gingivitis	2
localized moderate to severe periodontitis to generalized moderate gingivitis	2
generalized severe periodontitis to generalized moderate to severe periodontitis	2
localized moderate to severe periodontitis to generalized moderate to severe gingivitis	1
generalized moderate periodontitis to localized mild gingivitis	1
generalized moderate to severe periodontitis to localized moderate periodontitis	1
localized moderate to severe gingivitis to localized moderate gingivitis	1
generalized moderate periodontitis to localized moderate gingivitis	1
generalized moderate periodontitis to generalized mild to moderate gingivitis	1
localized mild periodontitis to localized severe gingivitis	1
localized moderate to severe periodontitis to generalized mild No disease	1
generalized severe periodontitis to generalized severe gingivitis	1
localized severe periodontitis to generalized mild to moderate gingivitis	1
localized moderate periodontitis to localized severe gingivitis	1
generalized periodontitis to generalized gingivitis	1
generalized mild periodontitis to localized severe gingivitis	1
localized mild periodontitis to localized moderate gingivitis	1
generalized mild to moderate periodontitis to localized mild to moderate periodontitis	1
localized moderate periodontitis to generalized severe gingivitis	1
generalized moderate to severe periodontitis to localized mild periodontitis	1
generalized severe periodontitis to localized mild to moderate periodontitis	1
localized moderate periodontitis to generalized mild to moderate gingivitis	1
generalized severe periodontitis to localized mild periodontitis	1
localized severe periodontitis to localized mild to moderate periodontitis	1
localized moderate to severe periodontitis to generalized severe gingivitis	1
generalized severe periodontitis to generalized moderate gingivitis	1
generalized moderate to severe gingivitis to generalized mild gingivitis	1
localized moderate periodontitis to localized moderate gingivitis	1
generalized severe gingivitis to localized mild gingivitis	1
generalized severe gingivitis to generalized mild to moderate gingivitis	1
localized periodontitis to generalized gingivitis	1
localized moderate gingivitis to generalized mild gingivitis	1

generalized gingivitis to localized mild to moderate gingivitis	1
localized gingivitis to localized severe gingivitis	1
localized moderate to severe periodontitis to generalized mild gingivitis	1
localized severe periodontitis to generalized severe gingivitis	1
localized mild periodontitis to generalized severe gingivitis	1
generalized moderate to severe periodontitis to generalized moderate gingivitis	1
Total	537

Table S4: Unknown periodontal disease change categories for which either disease type or severity information was not available from clinician-recorded diagnoses.

FROM disease type TO disease type	Number of patients
generalized mild No disease to generalized mild No disease	46
generalized moderate No disease to generalized moderate No disease	40
generalized gingivitis to generalized mild gingivitis	30
generalized mild to moderate periodontitis to generalized mild periodontitis	22
generalized moderate periodontitis to generalized moderate No disease	15
generalized No disease to generalized No disease	15
generalized mild gingivitis to localized mild No disease	9
generalized periodontitis to generalized moderate periodontitis	8
localized mild periodontitis to generalized moderate gingivitis	8
generalized mild periodontitis to localized moderate No disease	8
generalized gingivitis to generalized mild to moderate gingivitis	7
generalized moderate periodontitis to localized severe No disease	7
generalized mild to moderate periodontitis to generalized mild No disease	6
generalized mild to moderate No disease to generalized mild to moderate No disease	6
generalized gingivitis to generalized moderate gingivitis	6
generalized mild periodontitis to localized severe No disease	5
generalized mild periodontitis to generalized moderate No disease	5
generalized mild No disease to generalized mild periodontitis	5
localized mild No disease to localized mild No disease	5
localized moderate No disease to localized moderate No disease	5
generalized gingivitis to localized No disease	4
generalized mild periodontitis to generalized mild No disease	4
generalized moderate gingivitis to localized moderate No disease	4
generalized moderate periodontitis to localized moderate No disease	4
generalized gingivitis to generalized severe gingivitis	3
generalized gingivitis to generalized moderate periodontitis	3

generalized mild gingivitis to generalized moderate No disease	3
generalized moderate gingivitis to generalized moderate No disease	3
generalized mild periodontitis to localized mild No disease	3
generalized moderate No disease to generalized moderate periodontitis	3
generalized periodontitis to localized moderate periodontitis	3
localized gingivitis to generalized mild periodontitis	3
generalized moderate No disease to localized severe periodontitis	3
generalized mild No disease to generalized moderate periodontitis	3
localized gingivitis to generalized mild gingivitis	3
generalized moderate periodontitis to generalized mild No disease	3
generalized periodontitis to generalized mild periodontitis	3
generalized mild gingivitis to localized moderate No disease	3
generalized gingivitis to localized mild No disease	3
generalized periodontitis to localized severe periodontitis	3
generalized periodontitis to localized mild periodontitis	3
generalized mild No disease to generalized mild gingivitis	3
localized mild periodontitis to localized mild No disease	2
localized mild gingivitis to generalized mild No disease	2
localized gingivitis to generalized moderate gingivitis	2
localized severe periodontitis to localized severe No disease	2
generalized mild to moderate periodontitis to generalized mild to moderate No disease	2
generalized mild gingivitis to generalized mild No disease	2
generalized moderate periodontitis to localized mild No disease	2
generalized moderate No disease to generalized mild periodontitis	2
localized moderate periodontitis to generalized mild No disease	2
generalized periodontitis to generalized severe periodontitis	2
localized mild periodontitis to generalized mild No disease	2
generalized mild to moderate periodontitis to generalized moderate No disease	2
localized mild No disease to generalized mild gingivitis	2
generalized moderate to severe periodontitis to generalized moderate No disease	2
localized mild gingivitis to localized mild No disease	2
localized periodontitis to generalized moderate periodontitis	2
generalized mild to moderate gingivitis to generalized mild to moderate No disease	2
generalized No disease to generalized mild to moderate periodontitis	2
generalized moderate gingivitis to localized mild No disease	2
generalized No disease to generalized mild gingivitis	2
localized periodontitis to localized mild periodontitis	2
generalized mild No disease to localized moderate periodontitis	2

generalized gingivitis to localized moderate No disease	1
generalized mild to moderate No disease to localized mild to moderate periodontitis	1
generalized moderate to severe periodontitis to generalized mild gingivitis	1
generalized mild No disease to localized moderate gingivitis	1
localized No disease to generalized mild periodontitis	1
generalized moderate No disease to generalized severe periodontitis	1
localized moderate gingivitis to localized severe No disease	1
localized moderate No disease to localized moderate periodontitis	1
generalized periodontitis to localized No disease	1
generalized moderate No disease to localized severe No disease	1
localized mild to moderate periodontitis to localized severe No disease	1
generalized severe periodontitis to localized moderate to severe No disease	1
localized moderate periodontitis to localized moderate No disease	1
generalized mild No disease to localized severe periodontitis	1
generalized periodontitis to localized moderate No disease	1
localized periodontitis to localized severe periodontitis	1
generalized No disease to generalized mild periodontitis	1
generalized moderate periodontitis to generalized severe No disease	1
generalized mild No disease to generalized moderate gingivitis	1
generalized mild gingivitis to localized severe No disease	1
localized No disease to generalized gingivitis	1
localized moderate No disease to localized severe periodontitis	1
generalized moderate No disease to generalized mild gingivitis	1
generalized No disease to localized mild to moderate periodontitis	1
generalized periodontitis to generalized mild gingivitis	1
generalized periodontitis to generalized severe No disease	1
generalized severe No disease to generalized moderate No disease	1
generalized gingivitis to generalized No disease	1
generalized moderate periodontitis to generalized mild to moderate No disease	1
generalized moderate to severe periodontitis to generalized mild No disease	1
generalized moderate No disease to localized moderate gingivitis	1
localized mild periodontitis to localized severe No disease	1
generalized periodontitis to localized moderate to severe periodontitis	1
localized mild periodontitis to generalized mild to moderate No disease	1
generalized moderate to severe periodontitis to localized moderate to severe No disease	1
generalized mild No disease to localized moderate No disease	1
generalized periodontitis to localized severe No disease	1
generalized mild to moderate gingivitis to generalized mild No disease	1

generalized moderate to severe periodontitis to localized moderate No disease	1
generalized mild to moderate gingivitis to localized mild No disease	1
generalized severe No disease to generalized mild periodontitis	1
localized No disease to generalized mild No disease	1
localized gingivitis to localized mild periodontitis	1
localized gingivitis to localized No disease	1
localized gingivitis to localized moderate periodontitis	1
generalized gingivitis to generalized mild to moderate periodontitis	1
generalized moderate to severe No disease to generalized moderate to severe No disease	1
generalized mild gingivitis to localized mild to moderate No disease	1
generalized severe periodontitis to generalized moderate No disease	1
generalized periodontitis to generalized mild to moderate periodontitis	1
localized periodontitis to generalized mild gingivitis	1
localized periodontitis to generalized moderate gingivitis	1
localized gingivitis to localized severe periodontitis	1
generalized severe No disease to generalized severe No disease	1
localized periodontitis to generalized moderate No disease	1
generalized mild periodontitis to localized moderate to severe No disease	1
generalized gingivitis to localized moderate gingivitis	1
generalized mild No disease to localized mild periodontitis	1
generalized moderate gingivitis to generalized mild No disease	1
generalized No disease to localized severe periodontitis	1
localized moderate to severe periodontitis to localized severe No disease	1
generalized moderate to severe No disease to generalized severe No disease	1
localized mild periodontitis to localized moderate No disease	1
generalized moderate to severe No disease to generalized moderate periodontitis	1
localized No disease to localized periodontitis	1
Total	437
